# Organotypic brain slice cultures as a model to study angiogenesis of brain vessels

**DOI:** 10.3389/fcell.2015.00052

**Published:** 2015-09-02

**Authors:** Bianca Hutter-Schmid, Kathrin M. Kniewallner, Christian Humpel

**Affiliations:** Laboratory of Psychiatry and Experimental Alzheimer's Research, Department of Psychiatry and Psychotherapy, Medical University of InnsbruckInnsbruck, Austria

**Keywords:** organotypic slice, vibrosection, vessel, capillary, vascular, pericyte

## Abstract

Brain vessels are the most important structures in the brain to deliver energy and substrates to neurons. Brain vessels are composed of a complex interaction between endothelial cells, pericytes, and astrocytes, controlling the entry of substrates into the brain. Damage of brain vessels and vascular impairment are general pathologies observed in different neurodegenerative disorders including e.g., Alzheimer's disease. In order to study remodeling of brain vessels, simple 3-dimensional *in vitro* systems need to be developed. Organotypic brain slices of mice provide a potent tool to explore angiogenic effects of brain vessels in a complex 3-dimensional structure. Here we show that organotypic brain slices can be cultured from 110 μm thick sections of postnatal and adult mice brains. The vessels are immunohistochemically stained for laminin and collagen IV. Co-stainings are an appropriate method to visualize interaction of brain endothelial cells with pericytes and astrocytes in these vessels. Different exogenous stimuli such as fibroblast growth factor-2 or vascular endothelial growth factor induce angiogenesis or re-growth, respectively. Hyperthermia or acidosis reduces the vessel density in organotypic slices. In conclusion, organotypic brain slices exhibit a strong vascular network which can be used to study remodeling and angiogenesis of brain vessels in a 3-dimensional *in vitro* system.

## Introduction

The organotypic brain slice model resembles partly the *in vivo* condition of a high density cell system. In slices, individual cells are in close contact and do not lose density dependent regulatory mechanisms, 3-dimensional architecture as well as tissue specific transport and diffusion probabilities. The model has been introduced by Gähwiler and colleagues (Gähwiler and Hefti, 1984; Gähwiler et al., 1997), modified by Stoppini et al. (1991) and meanwhile used and characterized by several research groups including ours (Gähwiler et al., 1990; Ostergaard, 1993; Robertson et al., 1997; Ullrich et al., 2011; Daschil et al., 2015).

Brain capillaries constitute the blood-brain barrier (BBB) and innervate all areas of the brain. A first description of vasculature in organotypic brains slices has been given in 1975 (Wolff et al., 1975). Subsequently, Renkawek et al. (1976) characterized brain capillaries in organotypic cultures by relative unselective butyryl-cholinesterase stainings. We were one of the first to demonstrate that organotypic brain slices contain a strong network of laminin^+^ brain capillaries (Moser et al., 2003, 2004). Laminin is a well-established basement membrane marker which excellently stains the vascular structures of the brain. We demonstrated that capillaries survive in organotypic sections without any circulation (Moser et al., 2003). Although, the capillaries are not functional any longer and do not display any blood flow, it is likely, that they express and secrete a cocktail of different molecules which may influence other cells within the slices including nerve fiber innervations (Moser et al., 2003). Furthermore, we have recently shown that brain vessels in organotypic brain slices can re-grow between specific areas, when exogenously stimulated (Ullrich and Humpel, 2009). Meanwhile, brain vessels are well studied in organotypic cultures and particularly the neurovascular unit (NVU) is intensively explored in this complex 3-dimensional network (Morin-Brureau et al., 2013; Chip et al., 2014). The use of growth factors, especially vascular endothelial growth factor (VEGF) is important to study angiogenesis and revascularization in organotypic slices (Morin-Brureau et al., 2011). Recently, the interaction of vascular cells with astrocytes and particularly with pericytes within 3-dimensional organotypic slices came into intense investigation (Mishra et al., 2014). Zehendner et al. (2013) provided a detailed characterization of such a novel organotypic *in vitro* model of the NVU in the developing cortex.

Thus, there is clear evidence that organotypic brain slices are suitable to explore angiogenesis, vascularization or re-growth of vessels. It is hypothesized that *in vitro* vessels react upon stimulation with growth factors or pharmaceutical drugs and may represent a situation, which may also be found *in vivo*. The aim of the present work is to first summarize published experiments (including own data) regarding modulation of the vascular network in organotypic brain slices. Second, we add novel data and show for the first time how pericytes can be studied in such brain slices.

## Material and methods

### Organotypic slices and vibrosections

Adult or postnatal day 8–10 mice (C57BL/6N, Charles River, wild-type, WT) were used. Postnatal animals were rapidly sacrificed with a large scissor and adult animals were first injected with a lethal dose of thiopental. Then the head was quickly transferred in 70% ethanol, the brains dissected and sagittally cut. The brains were glued (Glue Loctite) onto the chuck of a water cooled vibratome (Leica VT1000A) and triggered close to a commercial shave racer. Under aseptic conditions, 110 μm thick vibrosections were cut and collected in sterile medium. The organotypic vibrosections were carefully placed onto a 0.4 μm membrane insert (Millipore PICM03050) within a 6-well plate (Greiner). Optional, slices were placed first onto a sterile 0.4 μm pore extramembrane (Millipore HTTP02500). Vibrosections (1–3 per well) were cultured in 6-well plates at 37°C and 5% CO_2_ with 1.2 ml/well of the following culture medium (Stoppini et al., 1991): 50% MEM/HEPES (Gibco), 25% heat inactivated horse serum (Gibco/Lifetech, Austria), 25% Hanks' balanced salt solution (Gibco), 2 mM NaHCO_3_ (Merck, Austria), 6.5 mg/ml glucose (Merck, Germany), 2 mM glutamine (Merck, Germany), pH 7.2. Vibrosections were incubated for minimal 2 weeks and medium was changed 1–2x per week. At the end of the experiment, vibrosections were fixed for 3 h at 4°C in 4% paraformaldehyde (PAF)/10 mM phosphate buffered saline (PBS) and then stored at 4°C in PBS/sodium acide until use. Alternatively, cortical brain pieces were cut into 400 μm thick sections using a MacIllwain tissue chopper and 6–8 slices were cultured on the membrane. Vibrosections or brain slices were either cultured with or without 100 ng/ml VEGF or FGF-2 for 2 or 4 weeks. Some slices were subjected immediately after dissection to 42°C (hyperthermia) or pH 6.0 (acidosis) overnight and then cultured for 2 weeks in normal medium. To study vessel re-growth, a cut was made with a scalpel through a whole vibrosection, starting from parietal cortex ending at the mesencephalon.

### Immunohistochemistry

Immunohistochemistry was performed as described in detail (Daschil et al., 2013). Brain slices were processed free-floating and were washed with PBS and incubated in PBS/0.1% Triton (T-PBS) for 30 min at room temperature (RT) while shaking. After incubation, the sections were blocked in T-PBS/20% horse serum (GIBCO Invitrogen)/0.2% BSA (SERVA) for 30 min at RT while shaking. Following blocking, brain sections were incubated with primary antibodies (collagen-IV, 1:500, abcam ab6586; alpha smooth muscle actin, αSMA, 1:1000, Novus Biologicals NB300-978; laminin, 1:500, Sigma L9393; PDGFRβ 1:250, Novus Biologicals NB110-57343) in T-PBS/0.2% BSA over 2–3 days at 4°C. The sections were then washed and incubated with fluorescent Alexa (-488, -546-; Invitrogen-Life Tech, Vienna, Austria) secondary antibodies in T-PBS/0.2% BSA for 1 h at RT while shaking. Finally, the sections were washed with PBS, then mounted onto glass slides and cover-slipped with Mowiol 4-88 (Roth, Austria). Some brain slices were processed using the chromogenic substrate diaminobenzidine DAB as described earlier (Ullrich et al., 2011).

Confocal microscopy was performed using a SP5 confocal microscope (Leica Microsystems, Wetzlar, Germany) with a HCX PL APO 63x/1.3 NA glycerol objective. Imaging was performed with an argon laser line for AlexaFluor 488, a DPSS561 nm laser for AlexaFluor 546 or thiazine red, and a HeNe 633 nm laser for AlexaFluor 647. Emission of each fluorophore was detected from 493 to 556 nm (AlexaFluor 488), 566 to 628 nm (AlexaFluor 546, thiazine red), and 638 to 750 nm (AlexaFluor 647). Images were acquired using the LAS AF acquisition software, version 2.1., and further processed with Huygens Deconvolution and Imaris V6.4 software.

### Western blot

Western blot analysis was performed as previously described by us (Hohsfield et al., 2014). Slices (adult and postnatal) were incubated for 2 weeks, and all slices from three wells were taken and pooled in an Eppendorf tube, then dissolved in 100 μl ice-cold PBS containing a protease inhibitor cocktail (P-8340, Sigma), homogenized using an ultrasonic device (Hielscher Ultrasonic Processor, Germany) and then centrifuged at 14,000 × g for 10 min at 4°C. Then, 20 μl of the extracts were loaded onto 10% Bis-Tris SDS-polyacrylamide gels, separated for 25 min at 200 V and finally electrotransferred to nylon-PVDF Immobilon-P^SQ^ membranes for 90 min at 30 V in 20% methanol blotting buffer. The Western Breeze Chromogenic System was used for the detection of specific proteins in cortical extracts. Briefly, blots were blocked for 30 min in blocking buffer, incubated with primary antibodies against PDGFRβ (1:2000) or actin (1:1000) at 4°C overnight, washed, and then incubated in alkaline phosphatase conjugated anti-rabbit IgG for 30 min. After washing, bound antibodies were detected using an enhanced chemiluminescence (ECL) system. As a control purified cultured brain capillary endothelial cells (BCEC) were isolated, extracted and loaded.

### Data analysis and statistics

The vascular density was counted in a 6 × 6 grid (see **Figure 2C**). Briefly, a digital picture was taken under the microscope at a 10x magnificiation. The digital picture was overlaid with a 6 × 6 grid using Photoshop (Adobe Photoshop Elements 2.0) and the number of all vessels crossing all lines was counted. Statistical analysis was performed by One-Way ANOVA and subsequent Fisher LSD *post-hoc* test. Statistical results were considered significant at *p* < 0.05.

## Results

### The vascular network in brain slices

**Figure 1:**
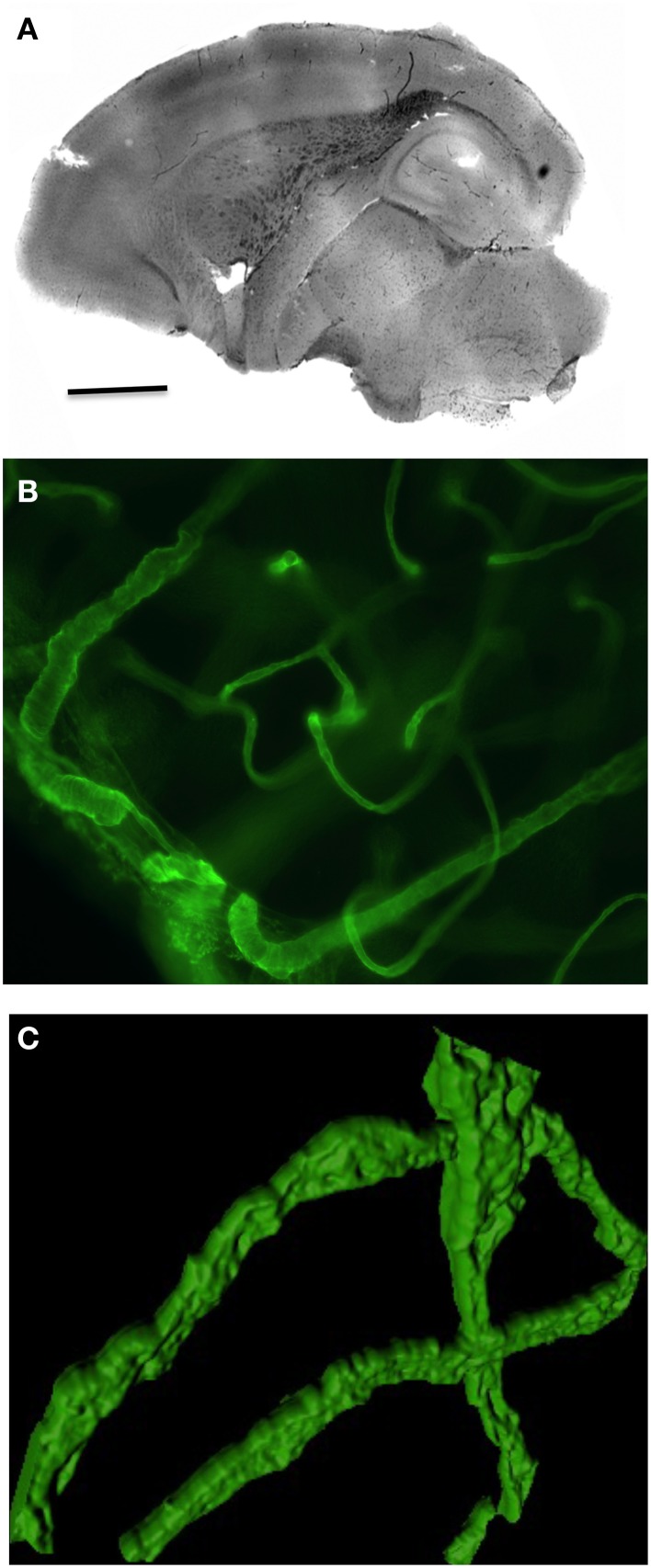
**Overview of vasculature in postnatal organotypic vibrosections**. Vibrosections (110 μm) were prepared from a 10 day postnatal wild-type mouse and cultured for 2 weeks, postfixed in paraformaldehyde and stained for collagen IV using a chromogenic DAB substrate **(A)** or fluorescence Alexa-488 **(B,C)**. Panel **(C)** shows a high power confocal microscopic picture. Scale bar in A = 1500 μm **(A)**, 17 μm **(B)**, 12 μm **(C)**.

**Figure 2:**
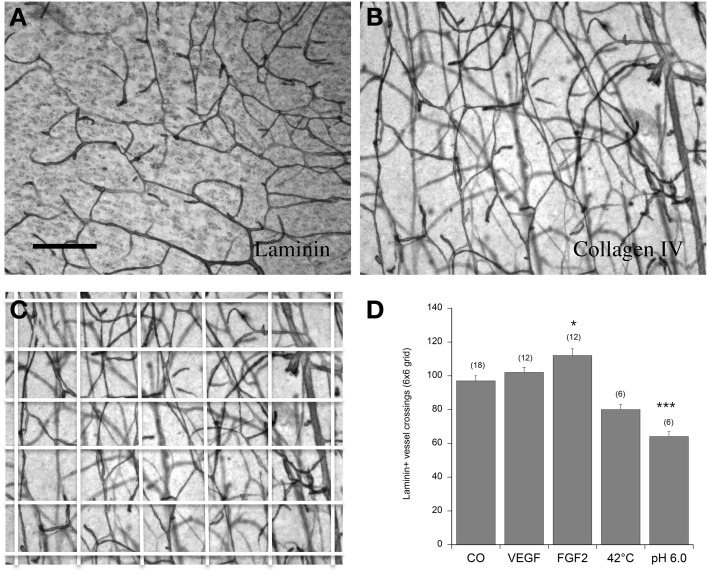
**Angiogenesis of vessels in the postnatal organotypic slice**. Brain slices from the cortex of 10 day old postnatal mice were cultured for 2 weeks either without (Co) or with 100 ng/ml VEGF or FGF-2 and then postfixed and stained for laminin **(A,D)** or collagen IV **(B,C)**. Some slices were incubated immediately after dissection at 42°C or at pH 6.0 overnight and then cultured for 2 weeks without any growth factors. The vessel density was quantified in a 6 × 6 grid **(C)**. Statistical analysis was performed by One-Way ANOVA with a subsequent Fisher LSD *post-hoc* test. Values are given as mean ± SEM; values in parenthesis give the number of analyzed slices. ^*^p < 0.05; ^***^p < 0.001. Scale bar in A = 90 μm **(A–C)**.

Organotypic brain vibrosections can be cultured from postnatal or adult mice and exhibit a strong vascular network all over the brain (Figure 1A). The vessels are well structured and express collagen IV (Figures 1B, 2B) as well as laminin (Figure 2A). The vessels are intact and represent tube-like formations as seen in the confocal microscope (Figure 1C).

### Effects on angiogenesis

In control brain slices, approximately 100 vessel crossings were counted in a 6 × 6 grid (Figure 2D). Incubation of postnatal brain slices with 100 ng/ml FGF-2 but not VEGF significantly increased the vessel density in the cortex (Figure 2D). When postnatal slices were pre-treated overnight with pH 6.0 (acidosis) but not with heat (42°C) the laminin^+^ vessel density significantly declined (Figure 2D).

### Effects on re-growth of vessels

**Figure 3:**
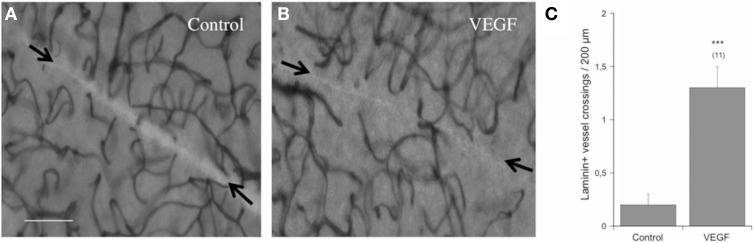
**Re-growth of vessels in the adult organotypic vibrosection**. Vibrosections were prepared from adult (6 month old) wild-type mice. A knife cut was made through the whole brain. Slices were then incubated for 4 weeks either without or with 100 ng/ml VEGF. Slices were then postfixed and stained for laminin using the chromogenic substrate DAB. The number of vessels crossing the cut (arrows in **A,B**) were counted and are given as crossings per 200 μm distance **(C)**. Statistical analysis was performed by an unpaired *T*-test with equal variance. Values are given as mean ± SEM; values in parenthesis give the number of analyzed slices. ^***^p < 0.001. Scale bar in A = 40 μm **(A,B)**.

In order to study re-growth, adult vibrosections were cut with a scalpel directly after transferring them on a semipermeable membrane. The number of laminin^+^ vessels crossing this cut was low (2 per field) after incubating for 4 weeks (Figures 3A,C). In contrast, when slices were incubated with 100 ng/ml VEGF, the crossing of vessels over the cut markedly increased (Figures 3B,C).

### Pericytes in brain slices

**Figure 4:**
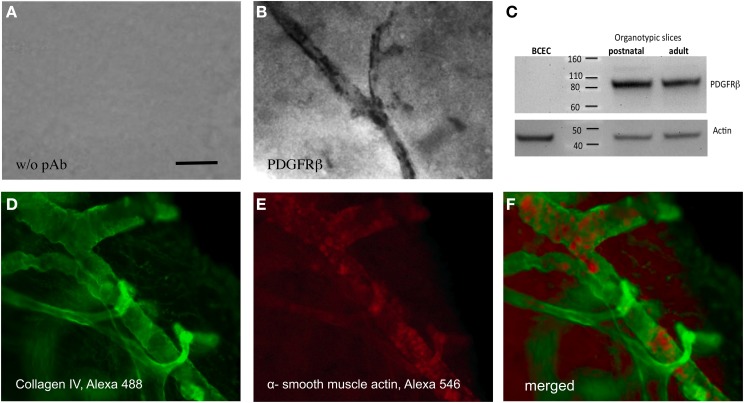
**Pericytes in adult organotypic vibrosections**. Vibrosections (110 μm) were prepared from the adult mouse, incubated for 2 weeks, then postfixed and stained for PDGFRβ **(A,B)**, collagen IV **(D)** or alpha-smooth muscle actin **(E)**. Slice extracts were subjected for a Western Blot and stained for PDGFRβ **(C)** showing a single band at approximately 80–100 kDa. As a control, extracts from cultured brain capillary endothelial cells (BCEC) or postnatal slices were analyzed. Loading control was performed by staining for actin **(C)**. Slices from panels **(A,B)** were stained using the chromogenic substrate DAB and slices from panels **(D–F)** by fluorescence (Alexa-488 for collagen IV, green and Alexa-548 for alpha-smooth muscle actin, red). Panel **(F)** shows a merged picture. Panels **(D,E)** were high power confocal microscopic pictures. Scale bar in A = 15 μm **(A,B)**, 7 μm **(D–F)**.

Pericytes specifically stained with an antibody against PDGFRβ, exhibit an intense staining along the vessel wall (Figure 4B). A control staining without antibody showed only background (Figure 4A). Western Blot analysis confirmed that postnatal as well as adult organotypic brain slices expressed an approximately 80–100 kDa PDGFRβ protein. As a control, purified cultured BCEC were negative. Actin was used as a loading control (Figure 4C). The collagen IV positive vessels co-expressed alpha-smooth muscle actin, another pericyte marker, as seen in the confocal microscope (Figures 4D–F).

## Discussion

In the present work we summarize published experiments and present examples of own data on exogenous modulation of the vascular network in organotypic brain slices. We show that brain slices exhibit a dense vascular network and are positive for laminin and collagen IV. We demonstrate that FGF-2 induces angiogenesis, however, that laminin^+^ staining is down-regulated by acidosis and that vessels can re-grow over a lesion site when stimulated by VEGF. Furthermore, we add novel data on the characterization of pericytes in organotypic brain slices and selective staining by PDGFRβ immunohistochemistry and Western Blot.

### The vascular network in organotypic brain slices

The brain is almost the only organ within the human body which depends on successive blood flow to ensure the continuous uptake of energy metabolites into neurons (Iadecola, 2004; Winkler et al., 2014a). In case of any interruption of the cerebral blood flow, brain function decreases immediately which leads to irreversible brain damage (Iadecola, 2004). Neurons are known to have high metabolic rates with only limited cellular reserves. Therefore, neuronal and cerebrovascular functions are tightly connected in the brain to guarantee appropriate vascular excess (Winkler et al., 2014a). Thus, almost every neuron within the human brain possesses its own capillary (Zlokovic, 2005). A proper function of the vascular system is essential for normal cerebral function and any alteration within this system leads to neuronal loss, stroke, vascular ischemia or vascular dementia, or Alzheimer's Disease (Ginsberg, 1990; Siesjoe, 1992; Hossmann, 1994).

In the last decades, several researchers investigated the function of the neurovascular system. Beside *in vivo* studies, *in vitro* techniques including organotypic brain slices became an own research focus for neuroscientists. Crain et al. (1982) established organotypic brain slices by using spinal cord-dorsal root ganglia. Moreover, in the following years, some alternative techniques came up comprising amongst others the roller-tube cultures, membrane cultures, and slices grown on culture dishes (Gähwiler and Hefti, 1984; Stoppini et al., 1991; Gähwiler et al., 1997). Recently, we developed a novel organotypic vibrosection model by culturing whole sagittal brain slices from postnatal rats for several weeks (Ullrich et al., 2011). Organotypic slices from different regions of the brain comprising striatum, hippocampus, cortex, cerebellum, and spinal cord are found to be a useful tool for physiological as well as pharmacological investigations (Gähwiler et al., 1997; Cho et al., 2007). Slices are easy to handle and maintain the cytoarchitecture of their originated brain region (Gähwiler et al., 1997; Ullrich et al., 2011). In the last years, *in vitro* studies using acute (dissected and analyzed the same day) brain slices were performed (Peppiatt et al., 2006; Fernández-Klett et al., 2010) However, acute brain slices do not represent the *in vivo* situation, they display an injured tissue with an improper BBB. Moreover, the constant cell death within acute brain slices accompanied with a severe BBB damage may disturb the mechanisms of the neurovascular coupling (Girouard and Iadecola, 2006; Filosa, 2010). Thus, there is clear need to culture slices for prolonged time to reduce any degenerative endogenous stimuli. The present model is such a chronic long-cultured system showing low astrogliosis or inflammation.

### Biomarkers to stain brain vessels

To investigate the vascular network, biomarkers like laminin or collagen IV are well established. Since decades, laminin is a well-known basement membrane marker which stains the vascular structures of the brain in an excellent manner (Eriksdotter-Nilsson et al., 1986; Jucker et al., 1996; Sixt et al., 2001). By using immunohistochemistry, several previous studies of our group displayed a strong network of laminin^+^ brain capillaries in organotypic brain slice cultures (Pirchl et al., 2006; Ullrich et al., 2011). Besides laminin, collagen IV is another basement membrane protein and is often used as biomarker for visualizing brain capillaries (Kefalides, 1966; Armulik et al., 2010; Daschil et al., 2015). In this study, we demonstrate that brain slices, which exhibit a dense vascular network are also positive for collagen IV. Using these biomarkers we determined the vessel density in our slices after treatment with different exogenous stimuli, such as growth factors, hyperthermia, or acidosis. It needs to be mentioned, however, that the immunostainings with a single marker only give some limited insight into the cellular processes. Thus, an increased laminin^+^ network could indicate that either the vessel density is increased and reflects angiogenesis or that only the vessel marker is up-regulated. The same is true if the vessel marker is decreased, which could reveal that the vessels are damaged and degenerate or that only the marker is down-regulated.

### Effects of growth factors VEGF and FGF-2 on vessel growth

Vessels are sensitive for degenerative stimuli, such as e.g., ischemia, trauma, stroke, or Alzheimer's Disease. Thus, there is increased importance to explore how vessels can be protected or how new vessels are formed. Several studies show that especially growth factors exhibit strong protective and growth-promoting effects (Kremer et al., 1997; Rosenstein et al., 1998). VEGF is the most potent factor controlling vascular function and formation to enhance the vasculature (Rosenstein et al., 1998; Carmeliet and Collen, 1999; Jośko et al., 2000). However, several other stimuli influence vessel growth. In our research group, we demonstrated a pro-angiogenic effect of thapsigargin in brain slices which is probably caused by an indirect stimulation of the VEGF expression (Ullrich and Humpel, 2009). In the present study, we show that VEGF has a potent effect on growth of vessels across a lesion site but not in an unlesioned slice.

A second potent vessel-promoting growth factor represents FGF-2 which is expressed in astrocytes. However, FGF-2 binds to fibroblast growth factor receptor-1 (FGFR-1) located on endothelial cells and regulates the survival of other angiotrophic factors (Garberg et al., 1998; Sobue et al., 1999). Several *in vitro* studies showed that decreased levels of FGF-2 lead to a malfunction of the BBB (el Hafny et al., 1996; Reuss et al., 2003). Bendfeldt et al. (2007) demonstrated for the first time that FGF-2 enhances the number of vascular structures in mouse brain in a concentration-dependent manner. Furthermore, FGF-2 promotes the function of the BBB by maintaining inter-endothelial tight junctions (Bendfeldt et al., 2007). In agreement with others, we show that FGF-2 significantly increased the vessel density in our organotypic brain slices.

### Effects of acidosis and hyperthermia

Acidosis is an important factor in ischemic brain pathologies including the damage of brain capillaries, NVU, and BBB (Siesjö, 1988; Pirchl et al., 2006). In general, acidosis is mainly provoked by increased CO_2_ levels in the tissue as well as dysfunctions within the brain metabolism leading to an abnormal accumulation of acids. As a result of hypercapnia, the pH level within the brain decreases to about 6.6 without affecting cell viability. However, in case of hypoxia and severe ischemia, anaerobic glycolysis leads to a pathological accumulation of acids, resulting in pH levels of about 6.0, which causes massive cellular damage (Rehncrona, 1985). Moreover, it is known that acidosis leads to an increase of iron-catalyzed production of reactive oxygen species by releasing iron from its binding partner ferritin or transferrin (Li and Siesjo, 1997). A few years ago, we (Pirchl et al., 2006) observed a decrease in laminin^+^ capillaries after incubating organotypic brain slices at low pH; this finding was confirmed in the present study.

Hyperthermia is another negative stimulus for the cerebral vascular system. Temperature rise, e.g., during fever, is a dangerous factor to cause brain damage (Fajardo et al., 1985; Ginsberg and Busto, 1998; Nybo et al., 2002). Our data show that, indeed, a rise of the temperature to 42°C overnight decreases the laminin^+^ vessel density, although not significant.

### Brain slices coupled to a blood-brain barrier

Isolated and cultured brain slices lack a BBB. In order to couple a synthetic BBB to slices Duport et al. (1998) developed a BBB *in vitro* model by co-culturing an endothelial cell monolayer upon a stationary organotypic slice culture. They (Duport et al., 1998) studied the attendance of tight junctions by using morphological analysis and neuronal activity using electrophysiological approaches. Additionally, they showed that dopamine and glutamate did not pass the synthetic BBB but L-DOPA entered the slices which demonstrated the selective permeability of the BBB. Despite the advantages of the system, a major drawback of this study was the number of unsuccessful cultures (error rate of about 25–30%). This model also failed in our hands due to its complexity and low stability of the confluent endothelial cell layer.

### Pericytes in organotypic brain slices

Pericytes are perivascular cells, uniquely located within the NVU and play an important role in the regulation of capillary blood flow, maintenance and formation of the BBB (Sagare et al., 2013; Winkler et al., 2014b). Along the arterial-venous axis, pericytes are mainly found on capillaries, pre-capillary arterioles, and post-capillary venules of many different organs (Dalkara et al., 2011). Especially in the capillary tube of the CNS, pericyte coverage of about 70–80% was found (Winkler et al., 2014b). During angio- and vasculogenesis, a cross-talk between endothelial cells and pericytes was observed leading to proliferation, migration, and attachment of pericytes to adjacent capillaries (Winkler et al., 2014b). Studies with pericyte-deficient mice (i.e., PDGFRβ transgenic mice) revealed that loss of pericytes is accompanied with vascular brain damage due to an increased BBB permeability (Dalkara et al., 2011; Winkler et al., 2014b). In organotypic brain slices, pericytes are not well studied. Zehendner et al. (2013) showed for the first time a detailed characterization of pericytes. Thereby, they observed that moderate hypoxia and inflammation caused caspase-3-mediated pericyte loss. Interestingly, Bell et al. (2010) determined that the loss of pericytes results in an impairment of the BBB and accumulation of several neurotoxic substances. Several biomarkers have been shown to be expressed by pericytes, such as e.g., PDGFRβ, NG2 (chondroitin sulfate), CD13 (alanyl (membrane) aminopeptidase), αSMA (alpha-smooth muscle actin), or desmin (Díaz-Flores et al., 2009; Armulik et al., 2010; Krueger and Bechmann, 2010). In the present study we show that within organotypic brain slices, pericytes can be selectively identified using PDGFRβ immunostaining and co-localize in αSMA^+^ vessels. Using Western Blot analysis, we confirm these data by showing PDGFRβ as a single band of approximately 80–100 kDa in adult as well as postnatal brain slices but not in endothelial cells.

### Organotypic brain slices as a screening model for angiogenic drugs

There is a clear need to develop fast and simple *in vitro* models for a high-throughput screening of pro-angiogenic factors or angiogenic inhibitors (Staton et al., 2004). So far the most useful angiogenic assays include *in vivo* Matrigel plug and sponge and corneal neovascularization, the chick chorioallantoic membrane and aortic arch assays, the *in vitro* cellular (proliferation, migration, tube formation) and organotypic (aortic ring) assays (Auerbach et al., 2003; Staton et al., 2009). Most pro- or anti-angiogenic drugs have been tested in co-cultures of endothelial cells and pericytes or smooth muscle cells forming a tubular network (Evensen et al., 2010, 2013; Fu et al., 2013; Wolfe et al., 2013). Organotypic brain slice cultures have to our knowledge not yet extensively used for screening pharmacological drugs, although this model is simple and provides a potent well preserved 3-dimensional capillary architecture. Using such slices we have recently tested different calcium channel blockers (e.g., nimodipine or nifedipine) and found strong pro-angiogenic activity (Daschil et al., 2015).

Taken together, our data summarize current literature and show examples of own data that organotypic brain slices of mice are an appropriate tool to study brain angiogenesis, vascularization, or vessel-re-growth. We provide evidence that screening of pharmaceutical drugs in such 3-dimensional brain slices may give insights into cellular and molecular processes of vessels, which may also play a role *in vivo*.

### Conflict of interest statement

The authors declare that the research was conducted in the absence of any commercial or financial relationships that could be construed as a potential conflict of interest.
